# Influence of Organic Acids on Diltiazem HCl Release Kinetics from Hydroxypropyl Methyl Cellulose Matrix Tablets

**DOI:** 10.4103/0975-1483.66793

**Published:** 2010

**Authors:** SB Sateesha, AJ Rajamma, MK Narode, BD Vyas

**Affiliations:** *Department of Pharmaceutics, Nargund College of Pharmacy, Bangalore 560 085, India*; 1 *Department of Pharmacognosy, KLES’s College of Pharmacy, Bangalore - 560 010, India*

**Keywords:** Diltiazem hydrochloride, organic acids, Peppas and Higuchi’s equations, pH independence, solubility

## Abstract

The matrix tablets of diltiazem hydrochloride were prepared by direct compression using hydroxypropyl methyl cellulose (HPMC) and various amounts (2.5%, 5.0%, 10% and 20%) of citric acid, malic acid and succinic acid. The characterization of physical mixture of drug and organic acids was performed by Infra-red spectroscopy. An organic acid was incorporated to set up a system bringing about gradual release of this drug. The influence of organic acids on the release rate were described by the Peppas equation: M _t_ /M_∞_ = Kt ^n^ and Higuchi’s equation: Q _t_ = K_1_t^1/2^. The addition of organic acids and the pH value of medium could notably influence the dissolution behavior and mechanism of drug-release from matrices. Increasing amounts of organic acid produced an increase in drug release rate, which showed a good linear relationship between contents of organic acid and drug accumulate release (%) in phosphate buffer, pH 7.4. The drug release increased significantly (*P* < 0.05) with use of succinic acid in tablet formulation. Increasing amounts of succinic acid above 10% produced decreasing values of *n* and increasing values of *k*, in a linear relationship, which indicated there was a burst release of drug from the matrix. Optimized formulations are found to be stable upon 3-month study.

## INTRODUCTION

Many drugs are weak bases or salts thereof and thus demonstrate pH-dependent solubility in the pH range of the GI tract. With controlled-release dosage forms, a possible pH-dependent release could result in *in vivo* variability and bioavailability problems.[1,2] Hence, pH-independent release of drug is desirable to better assure a reliable drug therapy and to build a greater control into a dosage form. Several attempts to overcome the problem of pH-dependent solubility of weakly basic drugs have been published. They are mostly based on the presence of acidic excipients such as water soluble or insoluble polymers or organic acids.[3,4] The effect of enteric polymers on release characteristics of weakly basic drugs with different p*K*a and aqueous solubilities at the pH of interest (7.4) were also investigated for their release from hydrophilic matrices.[5] These excipients either increase the release of drug from the delivery system by leaching out at higher pH values or keeping the pH low within the system in the intestinal pH range and thus the solubility of the drug high. One approach to overcome the problem of pH-dependent drug release is demonstrated in this paper, i.e., using common organic acids to create an acidic pH inside the polymer matrices.

Diltiazem hydrochloride is a calcium channel blocker presently considered an important drug for the treatment of hypertension. In the case of hypertension, successful treatment can be achieved only by maintaining blood pressure at a normal physiological level, and for this a constant and uniform supply of drug is desired. Diltiazem hydrochloride with all evident advantages proved to be a suitable candidate for development of a controlled-release dosage form, but the solubility of diltiazem hydrochloride is pH-dependent could result *in vivo* variability and bioavailability problems.[6] HPMC K-15M is employed to formulate controlled-release tablets of diltiazem hydrochloride since it could retard the release up to 24 h. The solubility of HPMC is pH-independent and forms a strong viscous gel on contact with aqueous media, which may be useful in controlled delivery of highly water-soluble drugs.[7,8]

## MATERIALS AND METHODS

Diltiazem hydrochloride was a gift sample from Anglo-French Drugs (Bangalore, India). Hydroxypropyl methyl cellulose (Methocel^®^, K-15M) was a gift sample from BPRL Private Ltd (Bangalore, India). The organic acids used were citric acid (Eros Pharma, Bangalore, India), malic acid (Lake Pharma, Bangalore, India), and succinic acid (Nice Chemicals, Mumbai, India). The lactose (Nice Chemicals, Mumbai, India), magnesium stearate (Loba Chemie, Mumbai, India), and all other reagents used in the study were laboratory grade.

### Formulation of tablets

Weighed quantity of drug, polymer, diluent, and one of the organic acids were mixed in geometric proportion. Amount of each ingredient was added as per Table 1, granules were prepared using water as a granulating fluid. Granules were dried and lubricated with 0.5% w/w magnesium stearate for 3 min. Then, 450 mg of lubricated granules were compressed with 10-station Rimek Minipress RSB-1 tablet punching machine using flat-faced punches (12 mm dia.). Characteristics of blend-like bulk density, compressibility index, and angle of repose were determined for each formulation. The dimensional specifications were measured using thickness gauge (Okimoto); weight variation test was conducted as per IP specifications. Hardness of the tablet was measured by using Pfizer hardness tester.

**Table 1:** Formulation ingredients (all quantities are in mg)

Formulation codes	Formulation ingredients						
	Diltiazem hydrochloride (mg)	HPMC (mg)	Lactose (mg)	Magnesium stearate (mg)	Citric acid (mg)	Malic acid (mg)	Succinic acid (mg)
F1	85	255	107.50	2.5	-	-	-
F2	85	255	96.25	2.5	11.25	-	-
F3	85	255	85.00	2.5	22.50	-	-
F4	85	255	62.50	2.5	45.00	-	-
F5	85	255	17.50	2.5	90.00	-	-
F6	85	255	96.25	2.5	-	11.25	-
F7	85	255	85.00	2.5	-	22.50	-
F8	85	255	62.50	2.5	-	45.00	-
F9	85	255	17.50	2.5	-	90.00	-
F10	85	255	96.25	2.5	-	-	11.25
F11	85	255	85.00	2.5	-	-	22.50
F12	85	255	62.50	2.5	-	-	45.00
F13	85	255	17.50	2.5	-	-	90.00

### Characterization by Infra-red spectroscopy

**Figure 1:**
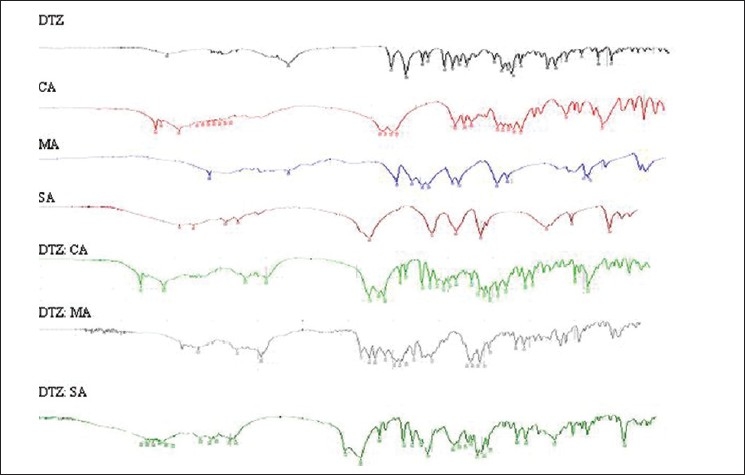
FT-IR spectra of diltiazem (DTZ) HCL, citric acid (CA), malic acid (MA), succinic acid (SA), and physical mixture of DTZ with CA, MA, and SA, respectively

Infra-red spectra for drug and organic acids were obtained on a infra-red spectrophotometer, model-8400S, Shimadzu Corporation, Kyoto, Japan, in the range of 4600–400 cm ^-1^ [Figure 1] with resolution of 4.0 cm ^-1^. KBr pellets were prepared by gently mixing the sample with KBr (1:100) and pressing the pellets at the pressure of 150 kg/cm ^2^ using pelletizer.

### Drug content determination

Tablet triturate equivalent to 85 mg of diltiazem hydrochloride was dissolved in 100 mL of 0.1 N HCl in volumetric flask, warmed at 50 °C for 30 min and filtered through 0.45 μm membrane filter. Sufficient dilutions were made from the filtrate to obtain 8 μg/mL solution. Absorbance of the resulting solution was measured using (UV1800 ENG240V shimadzu) spectrophotometer at 236 nm.

### Swelling behavior of matrix tablets

The extent of swelling was measured in terms of percent weight gain by the tablet.[9] The swelling behavior of formulation *F*_1_,*F*_4_,*F*_5_ and *F*_12_ were studied. One tablet from each formulation was kept in a beaker containing 100 mL of pH 7.4 phosphate buffer. At the end of 1 h, the tablet was withdrawn, soaked with tissue paper and weighed. Then for every 2 h, weights of the tablets were noted and process was continuous till the end of 12 h. The percentage weight gain by the tablet was calculated by formula

$$\mathrm{SI}\;=\;\left\{\left(M_{t\;}-\;M_{o}\right)/M_{o}\right\}\;\times \;100,$$

where SI is swelling index, *M*_t_ is the weight of tablet at time “*t*”, and *M*_o_ is the weight of tablet at time “*t*”=0.

### *In vitro* drug release studies

The *in vitro* dissolution studies were carried out by USP 24 dissolution apparatus type II paddle at 50 rpm. Dissolution test was carried out for a total period of 12 h using, 0.1 N HCl (pH 1.2) solution (900 mL) as dissolution medium at 37° 0.5° for first 2 h, and pH 7.4 phosphate buffer solution (900 mL) for rest of the of period. Five milliliters of the sample was withdrawn at regular intervals and replaced with the same volume prewarmed (37° ± 0.5°) fresh dissolution medium. The samples withdrawn were filtered through 0.45 μm membrane filter, and drug content in each sample was analyzed after suitable dilution. The actual content in samples was read from a calibration curve prepared with standard diltiazem hydrochloride.[10]

### Solubility determination

Solubility measurements of diltiazem hydrochloride were conducted in the pH range from 2.0 to 8.0 at 37°. An excess finely powdered drug was added to buffer solution of respective pH. The flasks were closed tightly and agitated at constant temperature (37° ± 1°) for 72 h. The solutions were filtered using 0.45 μm membrane filter and drug concentration in the filtrate was determined using UV-spectrophotometer. Solubility measurements of organic acids were also determined spectrophotometrically.[10–11]

### Kinetic analyses of dissolution data

The commonly adopted model for understanding release behavior of a drug from hydrophilic matrix is a simple exponential equation. The *in vitro* drug release data were fitted into Korsmeyer–Peppas equation[12] *M*_t_/*M*_∞_ = *Kt*^n^, where *M*_t_/*M*_∞_ corresponds to the amount of drug released at time t and after an infinite time, *K* is a constant related to the structural and geometric properties of the drug delivery system (tablet) and *n* is the release exponent related to the mechanism of the release. The *n* values used for the elucidation of the drug release mechanism from the tablets were determined from log cumulative percentage of drug released versus log time plots (i.e., log (*M*_t_/*M*_∞_ × 100) versus log *t*]. The release data were also analyzed as per Higuchi’s equation[13] *Q*_t_ = *K*_1_t^1/2^, where *Q*_t_ is the amount of drug released at time *t* and *K*_1_ is the diffusion rate constant. Table 2 shows an analysis of diffusional release mechanism obtained by various values of *r*^2^ and *n*.

**Table 2:** Data obtained from evaluation of tablets^*^

Formulation code	Hardness (kg/cm^2^)	Friability (%)	Drug content (%)	Cumulative % drug release at 12^th^ h
F1	6.0 ± 0.50	0.47 ± 0.02	97 ± 0.18	33 ± 0.60
F2	5.7 ± 0.29	0.65 ± 0.05	98 ± 0.08	48 ± 1.00
F3	5.3 ± 0.29	0.72 ± 0.07	97 ± 0.00	57 ± 1.00
F4	5.0 ± 0.00	0.72 ± 0.07	97 ± 0.08	64 ± 0.07
F5	4.3 ± 0.29	0.65 ± 0.08	97 ± 0.08	70 ± 1.00
F6	5.8 ± 0.29	0.73 ± 0.05	96 ± 0.20	46 ± 0.60
F7	5.3 ± 0.29	0.73 ± 0.05	99 ± 0.18	59 ± 1.00
F8	5.7 ± 0.29	0.78 ± 0.05	99 ± 0.18	65 ± 0.58
F9	4.3 ± 0.29	0.78 ± 0.05	99 ± 0.20	72 ± 1.30
F10	5.8 ± 0.29	0.67 ± 0.05	97 ± 0.18	49 ± 0.80
F11	5.3 ± 0.58	0.75 ± 0.10	96 ± 0.20	61 ± 1.50
F12	5.2 ± 0.29	0.70 ± 0.10	99 ± 0.08	77 ± 1.50
F13	4.3 ± 0.29	0.78 ± 0.07	97 ± 0.08	83 ± 0.58

All the values are given in ±SD (n = 3)

### Statistical analysis

The results are given as mean ± SD. The data were subjected to one way analysis of variance (ANOVA) for analyzing the statistical difference using the software PRISM (Graphpad, San Diego, CA). A confidence limit of *P* < 0.05 was fixed for interpretation of the results.

### Stability studies

To determine any change in *in vitro* drug release profile on storage, stability studies were conducted using a stability chamber, Fatha instruments (Hum 1017) for F _12_ formulation at 45° and 75% RH. The formulation was withdrawn after 4 weeks and evaluated for change in hardness, drug content and *in vitro* drug release pattern.

## RESULTS AND DISCUSSION

The solubility studies of diltiazem hydrochloride at different pH buffer solutions indicate that it has very low solubility at basic pH. Indeed, its solubility at pH 3.6 is 879.03 mg/mL, at pH 6.0 is 378.68 mg/mL, and at pH 7.4 is reduced to 71.42 mg/mL. Further, the *in vitro* drug release characteristics of formulation *F*_1_ (without solubility modifier), exhibited the good dissolution profile of the initial 2 h (0.1 N HCl), but slowest dissolution in basic medium (phosphate buffer pH 7.4). The fact may be due to high p*K*a (7.5) value of diltiazem hydrochloride. Hence, in order to modify dissolution profile the decreasing p*K*a value of the tablet core would be ideal. Hence, commonly available organic acids with low p*K*a value were selected, such as citric acid, malic acid, and succinic acid were added in different ratios in to the formulation [Table 1].

The FT-IR spectra of drug, organic acid and physical mixture (1:1) of drug with organic acid showed [Figure 1] no significance shift or reduction in intensity of peaks of drug. However, the IR spectra of physical mixture of drug with malic acid and succinic acid showed significance difference in the characteristic peaks of drug at 3450.77 cm ^-1^, but this is due to biotransformation of drug not by interaction with acids as it is confirmed.

**Figure 2:**
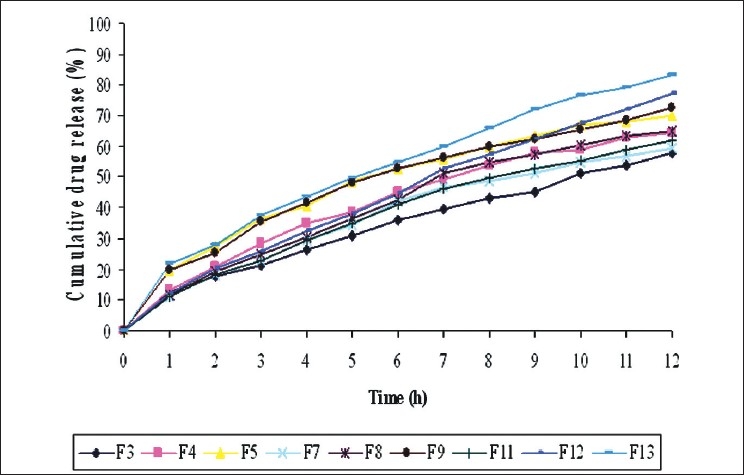
Comparison of *in vitro* release profiles of HPMC matrix tablets of diltiazem hydrochloride

The swelling index for HPMC was calculated with respect to time. It has been observed that the cumulative percent drug release decreases with increasing concentration of HPMC and swelling index. The reason attributed to this fact is slow erosion of the gelled layer from the tablets containing higher amount of HPMC[14–15]. The granules for tablet preparation were prepared according to the formula given in Table 1. Initially, tablets were prepared with a drug-to-polymer ratio of 1:1 and 1:2, water as a granulating agent. However, the drug release from these tablets was not uniform. Hence, the tablets with drug-to-polymer ratio of 1:3 were prepared to develop controlled drug delivery systems. The granules of different formulations were evaluated for angle of repose, loose bulk density (g/mL), tapped bulk density (g/mL), and compressibility index (%). All these results indicate that the granules possessed satisfactory flow properties and compressibility. The tablets of all the formulations were subjected to various evaluation tests. All the tablet formulations have shown acceptable pharmacotechnical properties even at varying concentration of organic acids and complied with the in-house specifications for weight variation, drug content, hardness, and friability [Table 2]. The dissolution studies have shown that all the formulations had released a high-cumulative amount of drug at the end of 12 h compared to *F*_1_ formulation [Figure 2]. This may be due to acidic microenvironment of the polymer followed by incorporation of organic acids[16]. Further to confirm the added organic acid resulted in an acidic microenvironmental pH within the core of the tablet, 0.15% of methyl red was added to the matrix to visually monitor the pH value within the tablet during the drug release. The experiments showed that the tablet slowly turned to yellow from periphery toward the centre at basic pH 7.4.

To know the mechanism of drug release from these formulations, the data were treated according to Higuchi’s (cumulative percentage of drug released versus square root of time), and Korsmeyer *et al*.’s. (log cumulative percentage of drug released versus log time) equations. The release rate kinetic data for the selected formulations can be seen in Table 3. As clearly indicated, the formulations did not follow a zero-order release pattern. Release of the drug from a matrix tablet containing hydrophilic polymers generally involves factors of diffusion. Diffusion is related to transport of drug from the dosage matrix into the *in vitro* study fluid depending on the concentration. As gradient varies, the drug is released, and the distance for diffusion increases. This could explain why the drug diffuses at a comparatively slower rate as the distance for diffusion increases, which is referred as square-root kinetics or Higuchi’s kinetics. In our experiments, the *in vitro* release profiles of drug from all the formulations could be best expressed by Higuchi’s equation, as the plots showed high linearity [Table 3]. To confirm the diffusion mechanism, the data were fit into Korsmeyer *et al*.’s equation. The formulations *F*_2_ to *F*_13_ showed good linearity (*R*^2^ = 0.983–0.996), with slope (*n*) values ranging from 0.515 to 0.778, indicating that diffusion is the dominant mechanism of drug release with these formulations. When plotted according to Korsmeyer *et al*.’s equation, formulation *F*_12_ also showed high linearity (R^2^ = 0.996), with a comparatively high slope (*n*) value of 0.778. This *n* value, however, appears to indicate a coupling of diffusion and erosion mechanisms, i.e., anomalous diffusion.

**Table 3:** T_50_%, r^2^ and n values for selected formulations*

Formulation code	T_50_%(h)	Correlation coefficient (Higuchi’s†)“r^2^”	Correlation coefficient (Peppa’s‡)“r^2^”	‘n’ Value (Peppa’s equation)	Mechanism of drug release
F3	10.52	0.978	0.990	0.628	Anomalous (non-Fickian)
F4	09.37	0.989	0.994	0.640	Anomalous (non-Fickian)
F5	08.57	0.995	0.991	0.519	Anomalous (non-Fickian)
F7	10.16	0.981	0.992	0.690	Anomalous (non-Fickian)
F8	09.23	0.975	0.994	0.710	Anomalous (non-Fickian)
F9	08.33	0.987	0.983	0.515	Anomalous (non-Fickian)
F11	09.83	0.981	0.996	0.703	Anomalous (non-Fickian)
F12	07.79	0.955	0.996	0.778	Anomalous (non-Fickian)
F13	07.22	0.986	0.985	0.580	Anomalous (non-Fickian)

† Higuchi’s equation, Q_t_ = *Kt*^½^

‡ Korsmeyer *et al*.’s equation, *M*_t_/*M*∞= Kt^n^

There was a moderate improvement of drug release could be found when citric acid or malic acid was incorporated in to the matrix, but drug release was slower as compared with that of succinic acid tablets. No significant difference (*P* < 0.05) in release rate was observed between tablets containing 2.5%, 5.0% or 10% of citric acid or malic acid. However, drug release increased significantly (*P* < 0.05) with 10% of succinic acid [Table 2]. Further increase in concentration of succinic acid above 10% results in decreasing values of *n* and increasing values of *k*, in a linear relationship, which indicates may be there was a burst release of drug from the matrix with the addition of succinic acid above 10%. On this basis 10% succinic acid, *F*_12_ was considered ideal for tablet formulation.

Stability studies of *F*_12_ formulation revealed that there was no significant change in hardness, friability, drug content, and dissolution profiles. Thus, *F*_12_ formulation was stable at different conditions of temperature. Therefore, the use of organic acids with low p*K*a value, in matrix tablets makes possible a controlled release of diltiazem hydrochloride whose solubility is pH dependent.
